# Reversible Diels–Alder Reactions with a Fluorescent Dye on the Surface of Magnetite Nanoparticles

**DOI:** 10.3390/molecules26040877

**Published:** 2021-02-07

**Authors:** Siyang He, Guido Kickelbick

**Affiliations:** Inorganic Solid-State Chemistry, Saarland University, Campus Building C4 1, 66123 Saarbrücken, Germany; he@dwi.rwth-aachen.de

**Keywords:** iron oxide, superparamagnetism, Diels–Alder reactions, organophosphonates, fluorescent dye

## Abstract

Diels–Alder reactions on the surface of nanoparticles allow a thermoreversible functionalization of the nanosized building blocks. We report the synthesis of well-defined magnetite nanoparticles by thermal decomposition reaction and their functionalization with maleimide groups. Attachment of these dienophiles was realized by the synthesis of organophosphonate coupling agents and a partial ligand exchange of the original carboxylic acid groups. The functionalized iron oxide particles allow a covalent surface attachment of a furfuryl-functionalized rhodamine B dye by a Diels–Alder reaction at 60 °C. The resulting particles showed the typical fluorescence of rhodamine B. The dye can be cleaved off the particle surface by a retro-Diels–Alder reaction. The study showed that organic functions can be thermoreversibly attached onto inorganic nanoparticles.

## 1. Introduction

Iron oxide nanoparticles play an important role in different disciplines due to their superparamagnetism, nontoxicity, and straightforward synthesis [1,2,3]. Many applications are arising due to their extraordinary properties in fields such as targeted drug delivery, biomedical imaging, hyperthermia, and even purification and separation technology [4,5,6,7,8]. One of the major issues is the surface functionalization of the particles with organic molecules allowing either a better dispersion in the targeted environment or an attachment of functional groups for specific interactions with the application environment [9,10]. Conventional preparation methods for Fe_x_O_y_ nanoparticles are precipitation/coprecipitation in solution, inverse micelle processes, and thermal decomposition. Precipitation processes in solution yield nanoparticles with relatively broad size distributions and their oxidation state is difficult to control [11]. Thermal decomposition of molecular precursors such as Fe(acac)_3_ allows size-controlled preparation of nanoparticles with narrow size distributions [12]. In all cases, stabilization of the particle surface is significant, and surface attachment of long alkyl chain carboxylic acids, amines, polymers, or inorganic coatings are general methods to achieve this. Exchange reactions of carboxylic acid or amine stabilizers with organophosphorus molecules are a simple route for organic post-functionalization of particles prepared by robust, high yield, and controlled synthetic procedures [13]. One interesting application of iron oxide particles is responsive inductive heating to external alternating magnetic fields in various environments. While this is currently mainly applied for hypothermia in biological environments, we also see some potential applications in molecular and polymer science [7,14,15,16,17,18,19,20,21].

**Figure 1:**
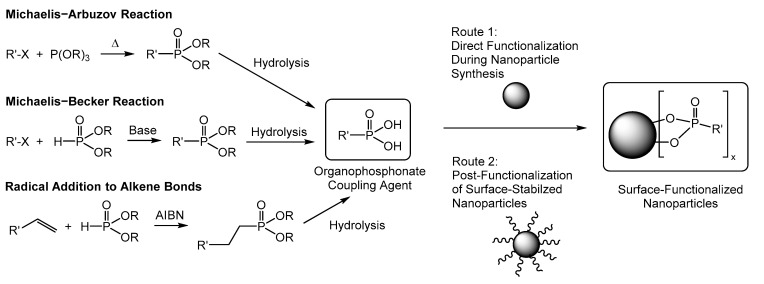
Synthesis of organophosphonate coupling agents and their attachment to the surface of metal oxide nanoparticles.

Particularly, the use of the superparamagnetic iron oxide particles as markers plays an important role in biomedical applications, due to low toxicity, potential magnetic targeting, and the ability to connect fluorescent molecules as probes for optical spectroscopy [22]. Various routes for introducing fluorescent dyes onto the surface of iron oxide nanoparticles are already described in literature. In principle, they can be divided into direct functionalization of the nanoparticles with fluorescent dyes that are modified with an anchor group, which allows attachments to the blank Fe_x_O_y_ surface, and methods that use core-shell particles with an additional inorganic shell attached on the particle surface [23]. Particularly, the use of phosphonate anchor groups attached to the iron oxide surface is advantageous because there are several methods to synthesize these molecules and to modify the surface of the iron oxide particles (Figure 1) [24,25,26,27,28,29].

Previous studies in our group showed that surface-functionalization of transition metal oxide nanoparticles with organophosphonates is a versatile route for fast and controlled surface functionalization [30,31,32,33,34,35]. Particularly, we were interested in modifying the surface of the nanoparticles with reversibly forming and cleavable organic bonds because these systems have a high potential in switchable applications such as self-healing materials. One of the most used reversible bond-forming systems is the Diels–Alder (DA) reaction between maleimide and furane groups due to its high efficiency, large temperature gap between the DA and retro-Diels–Alder (rDA) reaction, straightforward methods to synthesize functional molecules, and availability of spectroscopic methods to follow the DA/rDA reaction [36,37,38,39]. So far, only a few studies reported DA reactions on the surface of iron oxide nanoparticles. Maleki et al. used vinylsilane modified SiO_2_-coated Fe_3_O_4_ nanoparticle for stable covalent bonding of various cyclic organic compounds [40]. Hayes et al. used differential heating of magnetic nanoparticles for heteroplexed, time-controlled release of conjugated fluorophores from the nanoparticle surface [41]. In both studies, either DA bond formation or cleavage was investigated.

The aim of our study was to surface functionalize iron oxide nanoparticles with phosphonic acids bearing a maleimide functional group to perform a DA reaction with a furan-modified organic molecule. We chose a rhodamine B isothiocyanate derivative containing the complementary DA group of those on the iron oxide nanoparticles. The molecule thus serves as a fluorescent probe to investigate the extent of the DA/rDA reaction.

## 2. Results

### 2.1. Synthesis of Iron Oxide Nanoparticles

**Figure 2:**
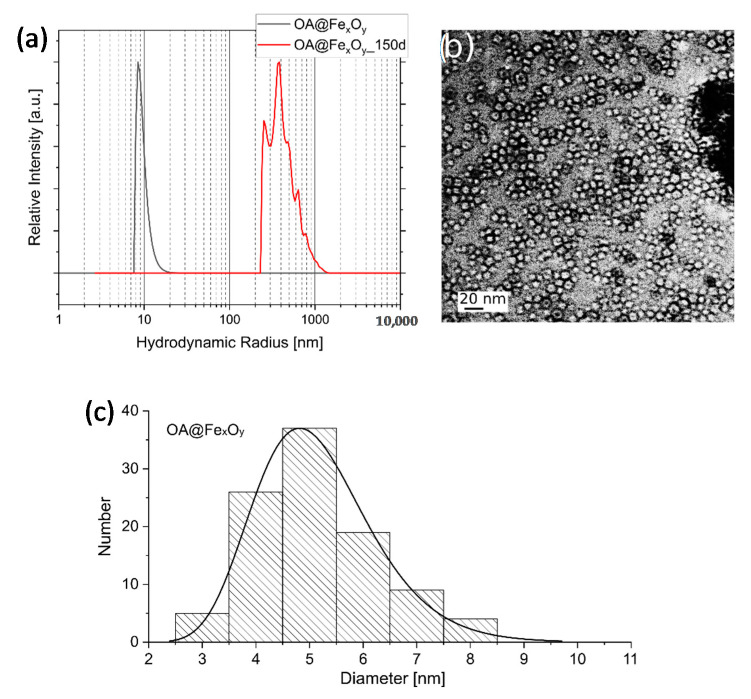
OA@Fe_x_O_y_ nanoparticles: (**a**) DLS curves in hexane, (**b**) TEM images, and (**c**) size distribution histograms obtained from 100 particles.

Oleic acid stabilized Fe_x_O_y_ nanoparticles (OA@Fe_x_O_y_) were prepared by thermal decomposition of Fe(acac)_3_ [39]. The particles obtained were dispersible in nonpolar solvents, such as hexane, and showed a hydrodynamic radius of 9.5(6) nm (Figure 2). However, they tended to agglomerate in this nonpolar solvent during storage, which should be considered during modification. Therefore, we recommend ethanol, chloroform, or solvent mixtures such as hexane/ethanol for long-term storage because this led to no such agglomerations in all functionalized nanoparticle cases. TEM images showed discrete particles that possessed a mean primary particle size of 5.16(11) nm. The observed discrepancy between dynamic light scattering (DLS) and TEM-averaged particle sizes resulted from the fact that only the particle core was observed in the TEM, whereas DLS-measured attached surface molecules and the solvent shell in addition to the core size. The attachment of oleic acid to the particle surface was detected by FTIR spectroscopy. The typical absorptions at 2924 and 2854 cm^−^^1^ result from the symmetric and asymmetric stretching vibrations of the methyl groups, and the band at 564 cm^−^^1^ can be assigned to the Fe-O stretching vibration. Signals at 1412 and 1520 cm^−^^1^ confirm the presence of carboxylate groups on the particle surface [39,42,43]. X-ray diffraction patterns proved that Fe_3_O_4_ was the only crystalline phase, and a crystallite size of 8.5(3) nm was calculated.

### 2.2. Preparation of N-(10-Maleimidodecyl)-Phosphonic Acid

**Figure 3:**
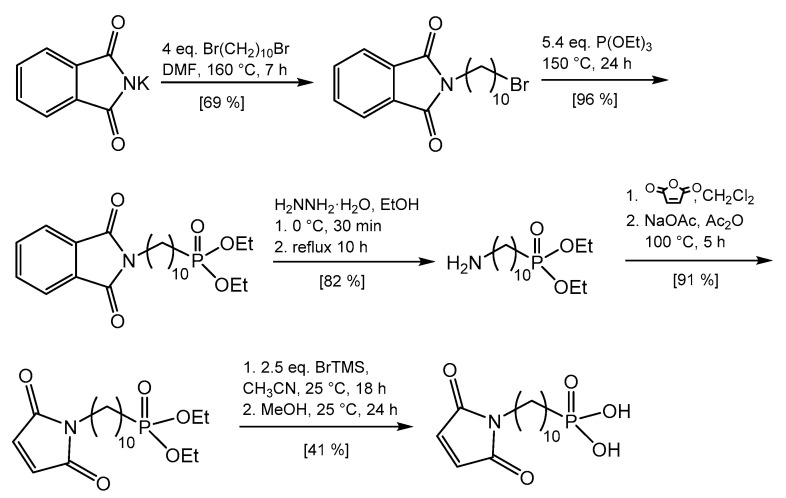
Synthesis of the maleimide phosphonate coupling agent.

*N*-(10-maleimidodecyl)-phosphonic acid was synthesized in a multistep approach developed in our group (Figure 3) [39]. Long alkyl chain spacers between the anchor group and the dienophile group are preferred because they provide higher degrees of freedom for the DA reaction. Due to the possible thermal polymerization of the maleimide group, a protection–deprotection synthesis is necessary to obtain the desired functionalized phosphonate coupling agent. Initially, *N*-(10-bromodecyl)-phthalimide was prepared by reacting potassium phthalimide with 1,10-dibromodecane. Then, the phosphonate ester group was introduced via a Michaelis–Arbuzov method by the reaction with triethyl phosphite. The desired primary amine was obtained by hydrazinolysis via Gabriel synthesis. In this step, it is recommended to use enough solvent to dissolve the primary amine as the main product and allow the byproduct phthalohydrazide to crystallize upon cooling, which could be easily filtered off. Then, the *N*-(10-maleimidodecyl)phosphonate diethyl ester was prepared using acetic anhydride as the dehydrating agent and sodium acetate as the catalyst. In the final step, the ester groups were cleaved with BrTMS, and subsequent hydrolysis with methanol gave *N*-(10-maleimidodecyl)phosphonic acid.

### 2.3. Maleimide-Functionalized Iron Oxide Nanoparticles

**Figure 4:**
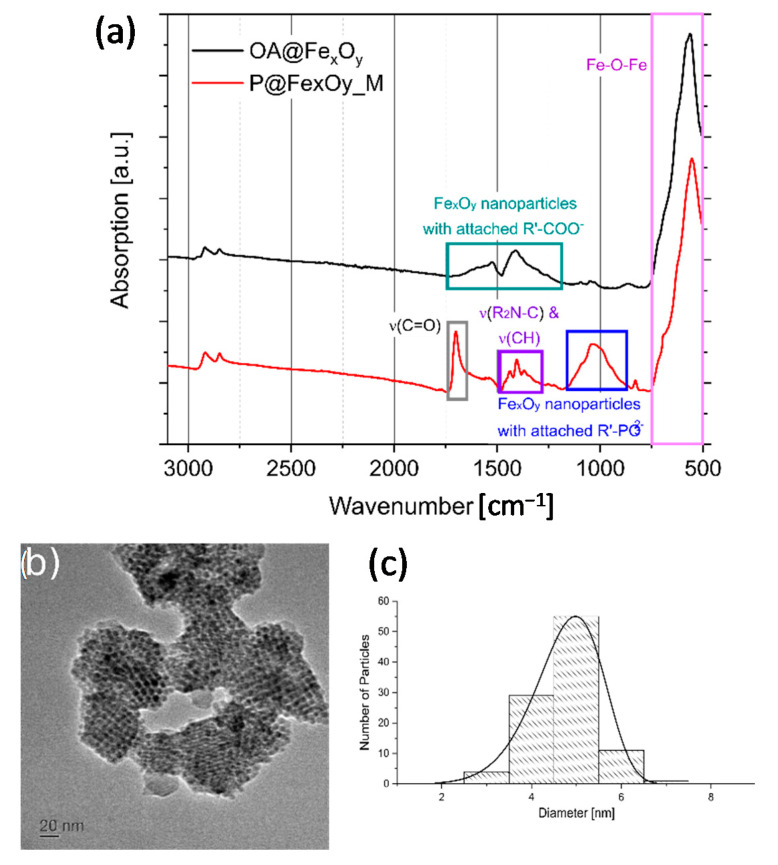
Maleimide-functionalized iron oxide nanoparticles: (**a**) FTIR spectra, (**b**) TEM image, and (**c**) size distribution histograms obtained from 100 particles.

Maleimide-functionalized nanoparticles were obtained through the substitution of the oleic acid on the OA@Fe_x_O_y_ nanoparticles with *N*-(10-maleimidodecyl)-phosphonic acid in a hexane—ethanol suspension. The phosphonic acid concentration in the solvent was optimized in a way that no excess of phosphonic acid was present. We controlled this by measuring the supernatant after modification with ^31^P NMR spectroscopy, which showed no signals for the phosphonic acid. The attachment of the phosphonic acid to the surface of the particles P@Fe_x_O_y_ was proven by FTIR spectroscopy (Figure 4a). Absorption bands for the attached phosphonic acid groups to the iron oxide surface and the maleimide carbonyl groups (1700 cm^−^^1^ for C=O, 1300–1500 cm^−^^1^ for R_2_N-C, 866–1150 cm^−^^1^ for P-O-Fe) were detected. Presence of oleic acid could not be excluded, because its typical absorption bands in the region of 1240–1670 cm^−^^1^ were located beneath characteristic vibration signals of the organophosphonate group.

DLS measurement of the maleimide-functionalized nanoparticles reveal a hydrodynamic radius of 22.5(6) nm. As expected, the size of the particles increased after the replacement of oleic acid against phosphonate groups on the surface, which also narrowed the size distribution. The enlargement can be explained by the different steric effects of oleic acid and the phosphonate groups. The oleic acid alkyl chain contains a cis-double bond, which prevents dense packing on the surface and sterically prevents interparticle agglomeration, while the synthesized phosphonates contain linear alkyl chains with a maleimide end group. The difference in chain composition results in a better interparticle agglomeration in case of maleimide-functionalized nanoparticles. TEM images of the nanoparticles support this argument, but also revealed that the size of the particle core and size distribution did not significantly change after modification (Figure 4b,c). The mean primary particle size of P@Fe_x_O_y_ was 4.78(71) nm.

Surface coverage of the P@Fe_x_O_y_ was calculated using the average TEM size of the particles (7 nm) and the mass loss from room temperature to 1000 °C from TGA measurement under pyrolytic conditions using a nitrogen atmosphere up to 900 °C followed by an oxidation step under synthetic air (N_2_/O_2_ 32:8) up to 1000 °C. During the oxidation, the pyrolytically formed graphitic carbon was completely removed due to the formation of CO_2_; Fe_3_O_4_ was oxidized to Fe_2_O_3_, which was proven by XRD measurements, and the phosphonate groups on the surface were most likely oxidized to phosphate. Using this method, a mass loss of 23.01% at 1000 °C was obtained for the P@Fe_x_O_y_ sample. Surface coverage was calculated assuming that the particles were dense spheres with a surface area of 154 nm^2^ (from TEM measurements). Equation (1) was applied to calculate the surface coverage of the phosphonic acids:

(1) $$\sigma =\frac{\Delta m}{M_{R}}\cdot \frac{1}{A}\cdot N_{A}\cdot 10^{-18}$$

where *σ*: surface coverage in molecules/nm^2^, Δ*m*: normalized mass loss in the TGA in %, *M_R_*: molecular weight of the part of the molecule that is decomposed, *A*: surface area of the particles in nm^2^, and *N_A_*: Avogadro constant. The anchor group of a phosphonic acid covers 0.24 nm^2^, which gives a maximum of around 4.2 molecules/nm^2^ for a completely covered surface [44]. Calculations on our samples reveal 3.8 molecules/nm^2^, which means that the surface of the particles would be densely covered with the phosphonic acid molecules. The calculated value is in good agreement with previously published surface coverages on metal oxide particles [45]. Although the equation takes into account oxidation of phosphonate groups to phosphate groups still attached to the particle surface, it does not take into account oxidation of Fe_3_O_4_ to Fe_2_O_3_, which leads to a mass gain. Therefore, the realistic value of surface coverage is a little smaller than the calculated value. From the ^31^P NMR spectrum of the supernatant after functionalization, it can be concluded that all phosphonic acid molecules were attached to the surface, either bonded or physically adsorbed. Equation (1), which is often used in literature, assumes a complete exchange of one surface attached ligand with another one. In our case, all carboxylic acids should be exchanged with phosphonic acid coupling agents. In Section 2.5, we will use elemental analyses of the particles to show that complete exchange does not occur, and therefore Equation (1) can only provide a rough estimate of the surface coverage. XRD measurements after functionalization showed that the crystalline core of the sample was still pure magnetite.

### 2.4. Modification of Rhodamine B Isothiocyanate with Furfurylamine

**Figure 5:**
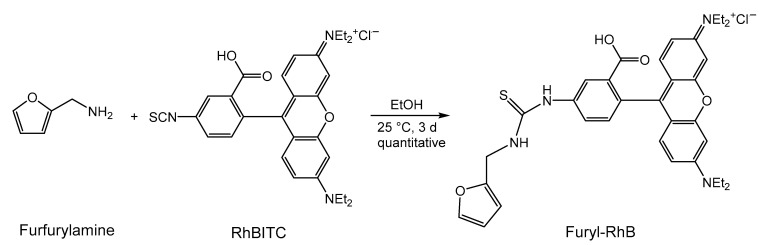
Addition of the furane ring as a diene component for the subsequent Diels–Alder (DA) reaction to rhodamine B isothiocyanate.

Rhodamine B isothiocyanate (RhBITC) was used as a fluorescent probe in our samples. This dye has excitation and emission maxima at 546 and 568 nm, respectively, corresponding to a Stokes shift of 22 nm. To react with the dieneophilic groups on the particle surface, it is essential to introduce a diene component into the dye molecule, and we performed the addition reaction with furfurylamine (Figure 5).

The completeness of the reaction was certified by the disappearance of the typical absorption peak of isothiocyanate group between 2175 and 1952 cm^−1^ in the FTIR spectrum. The product revealed the characteristic absorption band of the furan ring at 733 cm^−1^. The ^1^H and ^13^C NMR spectra also confirmed the addition reaction. The modified dye indicated a redshift of 3 nm in the excitation maximum to 546 nm and in the emission maximum to 571 nm in fluorescence spectroscopy.

### 2.5. Investigation of the Diels–Alder Reactions

**Figure 6:**
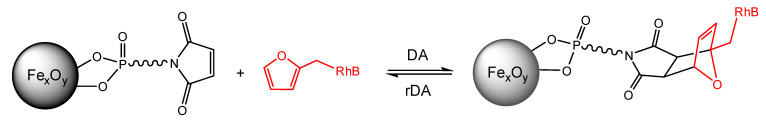
DA and retro-Diels–Alder (rDA) reaction on the surface of the iron oxide nanoparticles.

The aim of our study was to investigate the Diels–Alder (DA) and retro-Diels–Alder (rDA) reactions between the furan ring of furfuryl-RhB and the maleimide ring attached to the surface of the particles via the phosphonic acid group (Figure 6). In a preliminary study, we determined the principal possibility of DA reaction in solution between furfuryl-RhB and *N*-(10-maleimidodecyl)-phosphonate diethyl ester at room temperature. For this purpose, furfuryl-RhB and the phosphonic acid were mixed stoichiometrically 1:1 in an NMR tube containing CH_3_OD and the reaction was monitored by ^1^H NMR spectroscopy. The study revealed that the DA reaction proceeded very slowly at room temperature and the first endo-DA adduct was only detected after one month.

**Figure 7:**
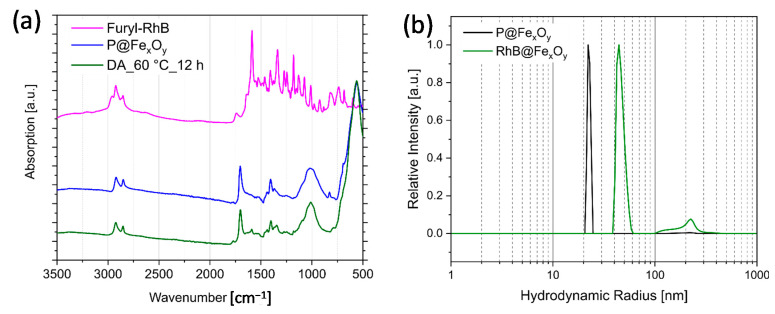
(**a**) ATR FTIR spectra of furfuryl-modified rhodamine b, phosphonate-modified Fe_x_O_y_ nanoparticles, and the DA product of both; (**b**) DLS of the particles before and after DA modification in EtOH.

**Figure 8:**
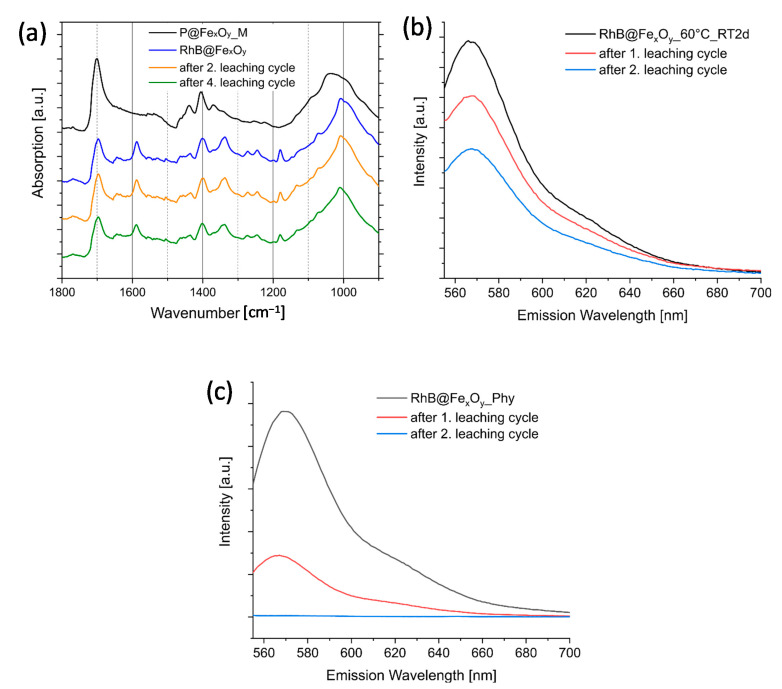
(**a**) FTIR overlay of the DA modified particles after specific leaching steps; (**b**) leaching followed with fluorescence spectroscopy of DA modified particles; and (**c**) those with the fluorescent dye only physically adsorbed at the particle surface.

Therefore, the study of DA reactions at the particle surface was carried out at higher temperatures. In a first approach, we allowed the P@Fe_x_O_y_ particles modified with maleimide groups to react with furfuryl-RhB in EtOH under reflux for 12 h. These reaction conditions only led to a low attachment of dye on the surface as detected by FTIR. One possible reason is that rDA reactions already occurred at this temperature. To optimize the reaction conditions for the DA reaction on the surface, we performed DSC measurements using P@Fe_x_O_y_ and furfuryl acetate as dienes without solvent. An optimized temperature of 60 °C was determined for the DA reactions on the surface. After reaction of the maleimide-modified iron oxide particles with furfuryl-functionalized rhodamine B at 60 °C for 48 h in chloroform, the successful DA reaction was confirmed by FTIR spectroscopy (Figure 7). Maleimide and furane rings show typically bands of their ring deformations at 696 and 721 cm^−1^, respectively, which can verify the successful DA reaction on the surface of particles [37]. In the case of iron oxide nanoparticles, however, these bands are hidden underneath the typical broad Fe–O bonds in the region of 560–580 cm^−1^ for Fe_3_O_4_ [46]. Nevertheless, the presence of bands at 1587, 1336, and 1180 cm^−1^ in the final product indicated a successful reaction. DLS studies after the DA reaction revealed a hydrodynamic radius of 46(3) nm, which can be attributed to the covalently attached large dye molecules. A small amount of additional agglomerates is visible at a hydrodynamic radius of 100–300 nm. After the DA reaction, the particles showed fluorescence of rhodamine B at 571 nm (λ_exc_ = 546 nm) (Figure 8b).

Carbon, hydrogen and nitrogen (CHN) analysis before and after the DA reaction allows an evaluation of the number of molecules attached to the surface of the maleimide-functionalized particles (Table 1). A good hint is the evaluation of the relative C:H:N values in the last column. The comparison of the values of the oleic acid and oleic acid modified particles shows the exact same C:H ratio. This observation provides evidence that this method allows an exact analysis of the organic content of the samples. For the evaluation of the attached coupling agents, we will only use the C:N value. The P@Fe_x_O_y_ particles should have a C:N ratio of 1:0.83 if a complete exchange of the oleic acid with the *N*-(10-maleimidodecyl) fragment would occur. The experimentally determined value was only 1:0.51, which means that around one third of the oleic acid ligands were not exchanged with the phosphonate coupling agents. After DA reaction, the fragment attached to the surface containing the furfuryl-modified rhodamine B should have a C:N value of 0.13. Experimentally, we determined a ratio of 1:0.06 after the DA reaction. If we set this into relation to the maleimide groups attached to the surface, we can calculate that only a minority of the groups reacted in the DA reaction. This is feasible based on the high steric demand of the furfuryl-modified dye molecule.

Leaching experiments can provide additional evidence regarding the dye binding onto the particle surface. Due to the hydrophobic dangling tails of the rhodamine B derivative after modification, a physical adsorption to the particle surface became possible. Nonbonded dye can desorb from the surface during purification of the particles in solvents. Therefore, we carried out leaching experiments in EtOH in which the dye-modified particles were ultrasonically dispersed in a solvent, allowed to stand for at least 24 h, magnetically separated, and the residual fluorescence of the particles was determined. If strong chemical bonds are present between the dyes and the surface of the particles, there should be no leaching or only a slight leaching of the attached dye. FTIR study revealed that for the DA reacted particles, the typical signals at 1587, 1336, and 1180 cm^−1^ of the rhodamine B derivative (Figure 8a) did not show any obvious changes even after the four leaching cycles. Furthermore, the typical fluorescence signal of rhodamine B at 571 nm was still detectable after several leaching cycles (Figure 8b). Particles which were just stirred in a dispersion containing a rhodamine b derivative that was not covalently attached were also treated with the same leaching conditions. These particles lost their fluorescence completely after the second leaching (Figure 8c), which was a strong sign for only physically adsorbed dye.

### 2.6. Investigation of retro-Diels–Alder Reactions at the Particle Surface

**Figure 9:**
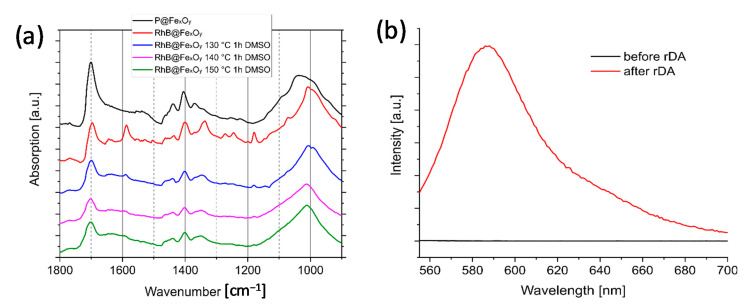
(**a**) FTIR spectroscopy of the RhB@Fe_x_O_y_ samples treated at rDA conditions; (**b**) fluorescence spectra before and after the rDA reaction at 130 °C.

The rDA reaction was investigated by heating an RhB@Fe_x_O_y_ dispersion in DMSO at 130, 140, and 150 °C for 1 h. The particles were magnetically decanted after the reaction, washed with ethanol, and the dried product was analyzed. FTIR spectra showed that the dye was rapidly separated from the particles with increasing temperature, which was the consequence of the rDA reaction (Figure 9a). At 130 °C, the dye molecules were almost completely removed from the particle surface. After the reaction at 140 °C, no more dye was detected on the particles. Additionally, the fluorescence band of the rhodamine B completely disappeared after heat treatment at 130 °C (Figure 9b). Since the onset of thiourea’s thermal decomposition is around 180 °C [47], cleavage of the thiourea bond is not feasible at the temperatures applied for the rDA reactions (≈120 °C). Hence, the reduction of the dye amount could only be explained by an rDA reaction.

## 3. Discussion

The controlled preparation of superparamagnetic Fe_3_O_4_ nanoparticles applying thermal decomposition of Fe(acac)_3_ yields well-defined particles with a diameter of around 7 nm and small size distributions. This literature-known synthesis is straightforward and provides a high yield, and forms ideal nanoparticles for further functionalization [12]. The particles obtained exhibit a hydrophobic surface and are therefore easily dispersed in organic solvents such as hexane. However, we observed agglomeration of the particles after prolonged storage. Sonication, change of solvents, or pH unfortunately did not facilitate the redispersion of the agglomerates. This means that freshly prepared oleic acid particles should be used immediately for further reactions. If the particle surface is to be functionalized with organic groups for further applications, replacement of the stabilizing carboxylic acid on the surface is necessary. Although this is an additional synthesis step compared to the simpler coprecipitation route, the extra effort is important for further studies because a well-defined Fe_3_O_4_ core allows straightforward systematic studies of surface-functionalization. Compared to the simple coprecipitation route, in which the particles have a broader size distribution and less control of crystallinity, which significantly affects the magnetic behavior, thermal decomposition provides well-defined building blocks [48,49,50].

In order to perform DA reactions on the surface, ligand exchange of the stabilizing ligands for functional ligands is necessary [51]. While a variety of molecules can be used for this exchange, organophosphorus molecules show a high exchange rate and strong binding to the surface [13,52,53]. The phosphonate group has several advantages over other anchor groups: (i) it can be easily incorporated into a molecule by various robust synthesis methods; (ii) it does not exhibit side reactions in solution, such as condensation reactions, which can occur with alkoxysilane anchor groups; and (iii) the synthesis can be monitored by ^31^P NMR spectroscopy. We succeeded in the synthesizing *N*-(10-maleimidodecyl) phosphonic acid, and finally obtained a molecule containing the phosphonate group with a C_10_ alkyl linker and a maleimide group as a dienophile for DA reactions. The applied five step synthesis also allows a simple exchange of the alkyl linker between the phosphonate and the maleimide group [39]. Certainly, it is also possible to synthesize an organophosphorus coupling agent with a furan functional group for the attachment to the iron oxide nanoparticle surface, but our experience is that the furan ring system tends to polymerize more readily in various synthetic reactions.

In the following exchange step of the carboxylic acid ligand with the organophosphorus ligand, it is important to know the extent of replacement of the ligands. An excellent method would be solid-state NMR in which we can detect carboxylate end groups of the original stabilizing ligands and phosphonate anchor groups of the organophosphorus molecules, but this is not possible with the paramagnetic Fe_3_O_4_ cores. Furthermore, it is only a qualitative method that shows whether a full exchange occurred, or whether the original stabilizing ligand is still present. Our IR spectroscopy measurements on the organophosphorus-modified particles showed only the presence of the phosphonates on the particle surface by detecting the typical signals of the phosphonate and the maleimide groups, but we cannot exclude that oleic acid was also still present. Literature studies illustrate that a full exchange of the stabilizing agents from synthesis is hardly possible [52,53]. A detailed study of the amount of stabilizers exchanged would be necessary, but is very demanding in terms of time and analysis. Therefore, the experimental conditions for the exchange should be chosen to ensure maximum exchange, which is why we stirred the suspension for several days. We used TGA to roughly estimate the number of ligands (surface coverage) after the exchange reaction. Assuming complete exchange of the carboxylic acid with the organophosphonate, a surface coverage of 3.8 molecules/nm^2^ was determined. However, this made it difficult to accurately calculate the maleimide groups on the nanoparticle surface.

We chose rhodamine B isocyanate as fluorescent molecules because of its simple modification with the commercially available furfurylamine in a quantitative addition reaction. Subsequently, the furfuryl-modified dye reacts with maleimide groups on the surface of the iron oxide nanoparticles. The DA reaction can be monitored by FTIR spectroscopy and the resulting particles show the expected fluorescence. Using optical spectroscopy, we were also able to ensure that the dye was truly bound to the surface by a covalent bond and not physically adsorbed. However, only a fraction of the maleimide groups on the surface of the dye reacted with the furan system, as demonstrated by elemental analysis. The reversibility of the DA reaction was demonstrated by the re-release of the dye under rDA conditions.

Further studies will show the reversibility of the reaction as well as the possibility of heat addition by external alternating magnetic fields, as recently reported by Hayes et al. [41].

## 4. Materials and Methods

### 4.1. Materials

Iron(Ⅲ) acetyl acetonate (>99.0%) was purchased from Acros Organics (Geel, Belgium), 1,2-dodecanediol (90.0%), oleylamine (70%), benzyl ether (98%), bromotrimethylsilanes (97%), n-butylamine (99.5%), rhodamine B isothiocyanate (mixture of isomers), and furfurylamine (99.0%) were purchased from Sigma-Aldrich (Darmstadt, Germany). Oleic acid (90%) and hexamethyl disilazane (98.5%) were purchased from abcr (Karlsruhe, Germany). Potassium phthalimide (>99%), maleic anhydride (≥99%), acetic anhydride (>99.5%), and sodium acetate (≥99%) were provided by Fluka (Charlotte, North Carolina, USA). Triethyl phosphite (98%), 1,10-Dibromodecane (97%), and hydrazine monohydrate (98%) were purchased by Alfa Aesar (Kandel, Germany). Magnesium sulfate (99%) was obtained from Grüssing (Hamburg, Germany) and zinc chloride (≥97%) from Carl Roth (Karlsruhe, Germany). All syntheses of phosphonic acid derivatives were carried out in absolute solvents purified with a solvent purification system (M. Braun Inertgas-Systeme GmbH, Garching, Germany) and under an argon atmosphere. Unless otherwise mentioned, all other chemicals were used without further purification.

### 4.2. Characterization Methods

Transmission electron microscope (TEM) measurements were carried on a JEOL JEM-2010 (JEOL, Tokyo, Japan). TEM samples were prepared by drop coating nanoparticle dispersions in methanol on the carbon coated copper grids (Plano S160-3). The particle size distributions were obtained from the TEM or SEM images by measuring 100 particles using the software ImageJ [54]. NMR spectra were recorded with an Avance III HD 400 MHz spectrometer (Bruker, Billerica, MA, USA) with 400.13 MHz for ^1^H NMR, 101 MHz for ^13^C NMR, and 161.98 MHz for ^31^P NMR. All NMR samples were prepared in CDCl_3_, D_2_O, or MeOD-d_4_. ATR-FTIR spectra were recorded using a Vertex 70 ATR-FTIR (Bruker Optics, Ettlingen, Germany) spectrometer equipped with a DIAMOND ATR-QL measurement cell in the range 4000–400 cm^−1^ with a resolution of 4 cm^−1^ using an average of 16 scans for both background and sample. Powder X-ray diffraction (PXRD) patterns were measured on a D8-A25-Advance (Bruker AXS, Karlsruhe, Germany) diffractometer in a Bragg-Brentano geometry using Cu Kα radiation. A 2θ range from 7° to 120° was recorded using a step size of 0.013° and a total measurement time of 1 h. The samples were prepared by drop coating the dispersed and homogenized nanoparticles in hexane onto glass sample holders. Sample compositions and crystallite sizes were determined from Rietveld refinements using TOPAS 5.1 [55]. The elemental analyses were conducted on an Vario Micro cube (Elementar, Langenselbold, Germany). Thermogravimetric analyses (TGA) were carried out on a TG F1 Iris (Netzsch, Selb, Germany) using a heating rate of 10 K/min in an atmosphere of synthetic air (N_2_/O_2_ 32:8, 40 mL/min). The samples were heated to 900 °C in open alumina crucibles and hold at this temperature for 15 min. DSC measurements were performed on a DSC 204 F1 Phoenix (Netzsch, Selb, Germany) in pierced aluminum crucibles under an atmosphere of N_2_/O_2_ 32:8 (100 mL/min) or N_2_ (100 mL/min) using a heating rate of 10 K/min. Dynamic light scattering (DLS) measurements were carried out at 25 °C using a compact goniometer (ALV, Langen, Germany). A scattering angle of 90° was used and the samples were prepared by dispersing the particles in methanol. The dispersions were ultrasonicated for 10 min, filtered through 0.45 µm PTFE filters, and equilibrated for 5 min before the measurements. Fluorescence spectroscopy was performed applying a FluoroMax 4 Spectrofluorometer (Horiba Scientific, Kyoto, Japan).

### 4.3. Syntheses

OA@Fe_x_O_y_ nanoparticles: The preparation of the iron oxide nanoparticles was based on a literature procedure [12]; 3.54 g (10 mmol) Fe(acac)_3_, 10.13 g (10.0 mmol) 1,2-dodecanediol, 10 mL oleic acid, and 10 mL oleylamine were dissolved in 100 mL benzyl ether in a 500 mL three-neck flask. The mixture was heated to 200 °C and kept at this temperature for 30 min. It was then further heated to 300 °C and kept at this temperature for 30 min. The hot plate was removed, and the mixture was cooled to room temperature. The particles were decanted using a magnet and washed three times with EtOH. The processed particles were stored in 96 mL of EtOH. For concentration determination, 5 mL of dispersion was removed; the particles were magnetically separated and dried in vacuo. The particle concentration and yield were calculated (47.1 mg/5mL; approximately 904.32 mg; TGA: 76.99% residual mass after the N_2_ segment to be counted as inorganic nuclei, ≈517 mg; with the molar mass of Fe_x_O_y_ 231.54 g/mol, the amount of substance is 9.02 mmol = 90%).

*N*-(10-Maleimidodecyl) phosphonic acid was synthesized following a previously described procedure [39].

Functionalization of the OA@Fe_x_O_y_ nanoparticles with phosphonic acid: 97.3 mg (0.3 mmol) of the prepared phosphonic acid were dissolved in a solvent mixture of 8 mL hexane and 2 mL EtOH; 17 mL of OA@FexOy dispersion in EtOH (160 mg particles) were added. It was stirred at room temperature for 60 h. The particles were magnetically decanted, washed with ethanol (3 × 15 mL), and stored in 20 mL EtOH. The particle concentration was determined with 5.7 mg/mL, yield: 114 mg. For further characterization the particles were dried in vacuo.

Preparation of furfuryl-RhB: Before synthesis, furfurylamine was dried over a molecular sieve (3 Å); 53.4 mg (0.1 mmol) RhBITC was dissolved in 30 mL of absolute EtOH under an argon atmosphere and 110 mg (11 eq., 1.13 mmol) furfurylamine were added. The mixture was stirred at room temperature for 72 h. The solvent was removed on a rotary evaporator and the excess furfurylamine (Sdp. 145 °C) was removed under high vacuum. The product was redissolved in 10 mL EtOH (0.01 mmol/mL).

ATR-FTIR: 3000–2827 (-CH_3_, -CH_2_-, O-H in COOH dimer), 1700 (C=O), 1645 (C=S), 1600–1400 (ring vibrations), 1380–1250 (υC-N, aromatic), 1230–1030 (υC-N, aliphatic) cm^−1^.

^1^H NMR (400 MHz, D_2_O): δ = 7.53 (dd, *J* = 1.9, 0.8 Hz, 1H, C5), 6.51 (dd, *J* = 3.3, 0.8 Hz, 1H, C1), 6.44 (dd, *J* = 3.3, 1.9 Hz, 1H, C2), 4.18 (s, 2H, C6).

DA reaction with molecular species: 1 mL of the above furfuryl-RhB solution (1 mmol furfuryl-RhB) was taken and EtOH was removed under high vacuum. Using *N*-(10-maleimidodecyl)-diethylphosphonate (3.8 mg, 0.01 mmol, compound 5), the two species were dissolved in MeOD-d4. After 4 and 30 days at room temperature, NMR spectra were recorded.

^1^H NMR (400 MHz, methanol-d4) δ = 7.49 (d, *J* = 1.8 Hz, 1H, H5), 6.70 (s, 2H, CH=CH in compound 5), 6.45 (d, *J* = 3.3 Hz, 1H, H1), 6.37 (dd, *J* = 3.4, 1.9 Hz, 1H, H2) ppm.

DA reaction at the particle surface: 3 mL solution of furfuryl-RhB in EtOH (0.01 mmol/mL, 0.03 mmol) was added to P@Fe_x_O_y__Agg (38.1 mg). Then, 2 mL of EtOH were added and the particles were dispersed by ultrasonication. Stirring was performed for 12 h under reflux, and after certain reaction time, the particles were purified and characterized. For purification, the particles were washed with EtOH and magnetically decanted. This procedure was repeated several times until the supernatant showed no detectable color. The prepared particles were stored in 5 mL EtOH.

RhB@Fe_x_O_y__60 °C and RhB@Fe_x_O_y__60 °C_RT2d: 4 mL of the P@Fe_x_O_y_ (22.8 mg) dispersion were withdrawn and EtOH was decanted using a magnet. The particles were redispersed in 5 mL of CHCl_3_, and 0.2 mL of solution of furfuryl-RhB in EtOH (0.01 mmol/mL, 2 × 10^−3^ mmol) was added. Stirring was performed at 60 °C for 12 h, and a fraction of particles were worked up and characterized as described above. The remaining particles were kept in the reaction mixture at room temperature for 2 days and then purified or characterized in the same manner. These two particles were each stored in 5 mL EtOH.

Leaching experiments: The synthesized RhB@Fe_x_O_y_ particles (about 2–3 mg) were ultrasonically dispersed in 5 mL EtOH and allowed to stand for at least 24 h. The supernatant was magnetically decanted. The particles were purified, dispersed in 5 mL EtOH, and characterized by fluorescence spectroscopy.

The rDA reaction at the particle surface: The synthesized RhB@Fe_x_O_y_ particles (about 2–3 mg) were dispersed in 10 mL DMSO and heated at 130, 140, and 150 °C for 1 h. Before and after the rDA reaction, the supernatants were characterized by fluorescence spectroscopy. Then, the particles were magnetically decanted and washed with EtOH (3 × 10 mL). After drying in high vacuum, the particles were characterized by FTIR and fluorescence spectroscopy. These particles were again dispersed in the DMSO supernatant from the rDA reaction containing the cleaved dye and allowed to stand at room temperature for several days. The particles were then processed and characterized by fluorescence spectroscopy.

## 5. Conclusions

Well-defined magnetite nanoparticles with an average diameter of 7 nm were synthesized by thermal decomposition of Fe(acac)_3_ in presence of oleic acid. The stabilizing ligands were successfully replaced with *N*-(10-Maleimidodecyl) phosphonic acid, which acts as a dienophile on the surface of the particles. CHN analysis revealed that about two thirds of carboxylic acids were exchanged by the organophosphonates. A furfuryl-functionalized rhodamine B dye was synthesized in high yields and reacted with the maleimide-functionalized nanoparticle surface. Only a fraction of the maleimide groups reacted in the DA reaction with the fluorophore. One reason for this is probably the high steric hindrance of the dye. Leaching experiments showed that the dye is covalently bound to the surface and can be cleaved from the surface under rDA reactions. Our fundamental study reveals that temperature-induced reversible covalent modification of iron oxide nanoparticles is possible. The temperature range of the DA/rDA reaction does not allow using the strategy shown for biological applications. However, we currently extend the use of these particles in environments where an induction heating with external magnetic fields would be an attractive alternative for classical ovens. Typical systems which are in our mind are particles with switchable surface polarity, for example in emulsion applications, or temperature triggered self-healing nanocomposites.

## Figures and Tables

**Table 1:** Results from elemental analysis of the samples compared with theoretical calculated values of fragments attached to the surface of the iron oxide particles.

Samples or Fragments	Results from CHN Analysis [Mass %]			Mass % Relation in the Samples or Fragments
	C	H	N	C:H:N
Oleic acid (C_18_H_34_O_2_) ^1^	76.54	12.13	0	1:0.16:0
*N*-(10-Maleimidodecyl) fragment (C_14_H_22_NO_2_) ^1^	71.15	9.38	5.92	1:0.13:0.83
Furfuryl-modified rhodamine B attached to *N*-(10-maleimidodecyl) fragment (C_34_H_37_ClN_4_O_4_S) ^1^	64.49	5.89	8.85	1:0.91:0.13
OA@Fe_x_O_y_	9.77	1.56	0	1:0.16:0
P@Fe_x_O_y_	14.43	2.32	0.74	1:0.16:0.51
RhB@Fe_x_O_y_	16.85	2.52	1.03	1:0.14:0.06

^1^ Results obtained from theoretical calculations.

## Data Availability

The data presented in this study are available in article.

## Sample Availability

Samples of the compound *N*-(10-maleimidodecyl) phosphonic acid are available from the authors.
